# The identification and correction of pseudohypercalcemia

**DOI:** 10.3389/fonc.2024.1441851

**Published:** 2024-10-31

**Authors:** Tongyong Yu, Xiaozhe Li, Beihui Huang, Jingli Gu, Junru Liu, Meilan Chen, Juan Li

**Affiliations:** The Department of Hematology, The First Affiliated Hospital of Sun Yat-sen University, Guangzhou, China

**Keywords:** monoclonal gammopathy, monoclonal immunoglobulin, globulin, albumin, hypercalcemia, pseudohypercalcemia, albumin-corrected calcium, albumin and globulin-corrected ionized calcium

## Abstract

**Background:**

We found that a subset of patients with monoclonal gammopathy exhibited hypercalcemia without apparent causes or clinical manifestations In a cohort of 289 monoclonal gammopathy patients, 2.4% presented with such atypical hypercalcemia, with one notable case exhibiting normal ionized calcium levels, suggesting the presence of pseudohypercalcemia.

**Objective:**

The aim of this study is to elucidate the factors contributing to pseudohypercalcemia in monoclonal gammopathy and to develop a novel globulin-corrected calcium formula for clinical application.

**Methods:**

This observational study enrolled 110 monoclonal gammopathy patients from our center. An additional 33 patients were recruited to validate the newly proposed formula. Univariate analysis identified potential risk factors. And multivariate logistic regression identified definitive influential factors. The determined influential factors were utilized to develop a formula by multiple linear regression, which was validated by a paired t-test and the Youden index.

**Results:**

This study found that globulin was a risk factor for pseudohypercalcemia. It revealed that pseudohypercalcemia should be considered in patients with globulin levels ≥61 g/L (*P*=0.014). Both albumin and globulin were confirmed as independent factors associated with bound calcium. Given that, we developed a formula to correct ionized calcium levels, which was consistent with ionized calcium tested by blood gas analysis. The diagnostic accuracy of the new formula (Youden index is 0.906) is better than the traditional formula (Youden index is 0.906). Interestingly, all monoclonal immunoglobulin types, except for light chains, showed an equal propensity to develop pseudohypercalcemia (*P*=0.306). It also showed a linear correlation between IgA, IgG, and IgM and bound calcium.

**Conclusion:**

This study confirmed that elevated globulin affects serum total calcium and offered the threshold of globulin ≥ 61 g/L in the differential diagnosis of peudohypercalcemia from hypercalcemia. The new formula based on albumin and globulin was developed, which was verified to be better than the traditional formula for correctly diagnosing hypercalcemia. In addition, we found that neither light chains nor heavy chains of monoclonal immunoglobulin alone can result in pseudohypercalcemia.

## Introduction

Monoclonal gammopathy (MG) is a group of diseases in which monoclonal immunoglobulin can be detected in blood and/or urine. They are synthesized and secreted by abnormal clonal plasma cells and/or B lymphocytes (1–4). Monoclonal gammopathy is a common condition associated with various diseases, such as multiple myeloma. Monoclonal gammopathy can lead to a variety of clinical manifestations, including the absence of clinical symptoms and a series of symptoms such as hypercalcemia, hyperviscosity, cyanosis, cryoglobulinemia, hemolysis, hemorrhage, anemia, renal insufficiency, and some related symptoms caused by monoclonal immunoglobulins deposited in local or systemic organs (5, 6).

Hypercalcemia is defined as serum total or corrected calcium ≥11 mg/dL (2.75 mmol/L) or ionized calcium ≥ 5.33 mg/dL (1.33 mmol/L) (7–11). Hypercalcemia is a common clinical manifestation of monoclonal gammopathy. The incidence of hypercalcemia in patients with multiple myeloma is 15% (12, 13). Hypercalcemia can cause symptoms in multiple systems and can be fatal in severe cases (14, 15). Moderate-to-severe hypercalcemia is an emergency in the department of hematology and requires aggressive treatment to reduce the calcium, such as loop diuretics, bisphosphonates, and glucocorticoids.

The etiology of hypercalcemia is mainly divided into tumors, which can cause hypercalcemia through bone destruction and the secretion of PTH-related peptide, renal dysfunction, endocrine diseases, drugs, familial hypocalciuric hypercalcemia, and granulomatous disease (16). Among them, bone destruction and renal dysfunction are the etiologies of hypercalcemia related to monoclonal gammopathy.

We found that a patient with monoclonal gammopathy had moderate-to-severe hypercalcemia through a blood biochemistry test. The patient was lacking an etiology of hypercalcemia due to the monoclonal gammopathy, such as bone destruction and obvious renal dysfunction (creatine 150 µmol/L, GFR 43 ml/(min×1.73 m^2^)), and the patient was also lacking clinical symptoms of hypercalcemia. For his hypercalcemia, we gave aggressive treatments to reduce the calcium, such as loop diuretics, bisphosphonates, and glucocorticoids, but the level of calcium decreased very slowly. For his high level of IgM, we performed plasmapheresis to reduce the monoclonal immunoglobulin. To our surprise, his serum total calcium decreased with decreasing monoclonal immunoglobulin (**Appendix Figure A**). We speculate that these patients may have pseudohypercalcemia due to markedly elevated monoclonal immunoglobulin, leading to elevated protein-bound calcium.

The case above is inconsistent with our previous clinical experience. In addition, we retrospectively analyzed the data of patients newly diagnosed with monoclonal gammopathy in our center from October 1, 2018, to February 22, 2021. Among 289 patients diagnosed with monoclonal gammopathy, 32 had hypercalcemia, accounting for 11%. Among these patients, seven had similar conditions, accounting for 2.4%. Neither clinical manifestations related to hypercalcemia nor bone destruction and no obvious renal insufficiency (GFR>30 ml/(min×1.73 m^2^)) (17) lead to the etiology of hypercalcemia related to monoclonal gammopathy. Therefore, the reason for hypercalcemia in these patients is worth discovering.

According to the literature, 99% of the calcium in the body is stored in the bones and teeth in the form of calcium phosphate and calcium carbonate. The remaining 1% of calcium exists in the blood, which is called serum total calcium (18). Serum total calcium is composed of anion-coupled calcium, ionized calcium, and bound calcium, of which ionized calcium accounts for approximately 45%, 45% is bound to protein, and 10% is coupled to inorganic or organic anions (19). In other words, serum total calcium is basically composed of bound calcium and ionized calcium. Bound calcium is calcium that is bound to proteins, mainly albumin. Because its affinity for globulin is relatively weak, only 19% is bound to globulin (20, 21). However, the influence of globulin on calcium is not clear. Ionized calcium is a calcium with physiological significance that can cause a series of clinical symptoms, is the index that truly reflects the active calcium level in the body (22–24) and is the gold standard for the diagnosis of hypercalcemia.

To our surprise, one of the seven patients above was found to be normal in ionized calcium by an ionized calcium test, which indicates that some patients with monoclonal gammopathy have pseudohypercalcemia. Pseudohypercalcemia is hypercalcemia with normal or low ionized calcium, which means high serum total calcium but normal or low ionized calcium (25). Serum total calcium is widely used as an indicator of the calcium concentration for diagnosis and clinical monitoring because the measurement of serum total calcium is more convenient and can be used on a large scale. However, it cannot truly reflect the physiologically active calcium level in the body because it is greatly affected by the albumin level and pH in the blood (26, 27). The measurement of ionized calcium depends on blood gas analysis, which greatly increases the cost of detection, requires strict sample processing, and requires drawing arterial blood, which increases the pain of patients, all of which limit the application of ionized calcium in the clinic (16, 20, 26, 28).

In the past, scientists have paid attention to the effect of bound calcium on serum total calcium. As a result of the limits of application of ionized calcium and the main components of bound calcium being albumin-bound calcium, scientists took advantage of more accessible parameters (serum total calcium, albumin) to establish the formula of albumin-corrected calcium, which is albumin-corrected calcium (mmol/L) = serum total calcium (mmol/L) − 0.025 × albumin (g/L) + 1 (mmol/L) (29–31), which is named Payne’s formula. They hoped to attenuate the effect of albumin on serum total calcium, reflecting physiologically active calcium, with this formula. However, the application of albumin-corrected calcium in various patients is controversial, and it will underestimate or overestimate the actual active calcium in the body (7, 9, 20, 22, 27, 32, 33).

According to the results of previous studies, albumin-corrected calcium cannot completely and comprehensively correct pseudohypercalcemia in patients with monoclonal gammopathy. Although the affinity of calcium for globulin is relatively weak, globulin also plays an important role in the state of calcium (7, 34). Meanwhile, we have found several case reports of pseudohypercalcemia in patients with monoclonal gammopathy (**Appendix Table A**) (7, 23, 25, 35–41). The type of monoclonal immunoglobulin (MIg) in these patients with pseudohypercalcemia includes IgA, IgM, and IgG, and the relationship between globulin and calcium needs further exploration.

Based on previous studies, we retrospectively analyzed the data of 289 patients with monoclonal gammopathy in our center. By multivariate logistic regression analysis, we found that globulin was an independent factor affecting serum total calcium (**Appendix Table B**). There was a linear correlation between globulin and albumin-corrected calcium (r=0.325, *P*=0.000) (**Appendix Figure B**). In conclusion, previous studies and retrospective studies have indicated that globulin still has a great effect on albumin-corrected calcium.

Physicians have always ignored the effect of globulin on calcium but have paid more attention to the effect of albumin in various diseases that cause large fluctuations in albumin. Many studies have proposed a formula for albumin-corrected calcium (42). Because of the few diseases that can cause large fluctuations in globulin and the weak affinity between globulin and calcium, the effect of globulin on calcium has not received enough attention (11, 20, 43). However, monoclonal gammopathy is one of the diseases that can lead to fold-increased globulin, and hypercalcemia is the main clinical feature of monoclonal gammopathy. As a result, the incidence of pseudohypercalcemia caused by hyperglobulinemia is relatively high. Therefore, the formula for albumin-corrected calcium cannot be used in the clinic in these cases. However, there are few case reports about the influence of globulin on calcium thus far and no studies about a formula for globulin-corrected calcium.

Therefore, this study evaluated the effect of globulin on calcium and analyzed the relationship between the type of monoclonal immuglobulin and pseudohypercalcemia and related factors of pseudohypercalcemia by collecting data from a relatively large sample with monoclonal gammopathy. We put forward a cutoff level of globulin that may lead to pseudohypercalcemia for the first time. Meanwhile, we tried our best to explore a formula for globulin-corrected calcium, which is suitable for patients with monoclonal gammopathy and will help physicians conveniently estimate the correct level of calcium. It is beneficial to avoid unnecessary interference, make diagnoses, and make the best choices for management.

## Methods

### Study design and patient selection

We performed a unicentric, observational study utilizing the digital medical records of 110 patients newly diagnosed with monoclonal gammopathy at the First Affiliated Hospital, Sun Yat-sen University, between February 23rd, 2021, and January 31st, 2022, as a training set. Subsequently, data from an additional 33 patients were collected to constitute a testing set. Eligibility for inclusion was based on the following criteria: a confirmed diagnosis of monoclonal gammopathy with a positive result from either serum immunofixation electrophoresis or Bence-Jones protein electrophoresis in serum or urine; first-time hospitalization at our center; and completion of a blood gas examination to assess ionized calcium levels. Exclusion criteria included missing vital data, presence of other solid tumors, hypercalcemia attributable to endocrine disorders, medication, granulomatous diseases, or rare genetic conditions. Ethical approval for this study was granted by the institutional review board (the number is [2022]471).

### Groups classification

Participants were categorized into three groups based on serum total calcium and ionized calcium test: the pseudohypercalcemia group, the actual hypercalcemia group, and the nonhypercalcemia group. The pseudohypercalcemia group comprised patients with albumin-corrected calcium ≥2.75 mmol/L and ionized calcium < 1.33 mmol/L (7–11, 25). The actual hypercalcemia group included patients with albumin-corrected calcium ≥2.75 mmol/L and ionized calcium ≥1.33 mmol/L (7–11, 25). The nonhypercalcemia group was defined by albumin-corrected calcium < 2.75 mmol/L and ionized calcium <1.33mmol/L (7–11). Comparative analyses were performed to identify factors influencing serum total calcium between the pseudohypercalcemia and actual hypercalcemia groups, and factors affecting bound calcium levels between the pseudohypercalcemia and nonhypercalcemia groups (**Appendix Table C**).

### Data collection

Clinical and demographic data were collected in this study, encompassing sex, age, diagnosis, serum total calcium level, bone destruction, specific disease type, creatinine, albumin, globulin, monoclonal immunoglobulin type, pH. Fasting venous blood samples were obtained for serum total calcium measurement, avoiding muscle contraction and minimizing tourniquet use during collection (27, 30, 33) Arterial blood was drawn for blood gas analysis, using a standard BD arterial blood collection device containing calcium-balanced heparin, in accordance with International Federation of Clinical Chemistry recommendations (IFCC) (20). Ionized calcium was detected by a GEM3000 blood gas analyzer. Creatinine, albumin, globulin, and serum calcium levels were measured using standardized techniques. Patients with a pH outside the reference interval of 7.35–7.45 were excluded to mitigate potential biases in calcium measurements.

### Statistical analysis

Data were analyzed using SPSS 22.0. Quantitative data are expressed as the mean and standard deviation fornormal distributed data or median and range for non-normally distributed data. Qualitative data are presented as frequencies and percentages. Statistical tests included t test, Mann−Whitney U test and chi-square test. Univariate analysis identified potential risk factors, with variables significant at *P <*0.1 advancing to multivariate logistic regression for determining influential factors. The receiver operating characteristic (ROC) curve was employed to establish the optimal cutoff for differentiating pseudohypercalcemia from actual hypercalcemia. The figures were generated by GraphPad Prism 7.0. All statistical tests were two-sided with *P <*0.05 considered statistical significance.

### Formula establishment and validation

Multiple linear regression was utilized to develop formulas correcting ionized calcium based on albumin, globulin, and serum total calcium as independent variables, with ionized calcium as the dependent variable. The diagnostic performance of the corrected ionized calcium was compared to direct measurements using a paired t-test and the Youden index.

The calcium corrected by the formula was assigned to be either true positive, true negative, false-positive or false negative (**Appendix Table D**). Then, the values were applied for determination of the following tests (Formula A1-A3).


Formula A1 Sensitivity %=True Postive ÷ (True Positive+False Negative)×100%

Formula A2 Specificity %=True Negative÷(True Negative+False Positive)×100%

Formula A3 Youden Index=(specificity+Sensitivity)−1

## Results

### Patient characteristics

The baseline characteristics of the 110 patients with monoclonal gammopathy included in our training set are presented in **Table 1**. The cohort had a mean age of 60 ± 11.51 years with an equal male-to-female ratio. The distribution of monoclonal immunoglobulin types was as follows: IgA in 30 patients, IgG in 46, IgD in 6, IgM in 4, free light chains in 23, and biclonal gammopathy in 1patient. Notably, 61.8% of these patients suffered from multiple myeloma (MM). Among all of the patients, 21 (19%) were diagnosed with hypercalcemia according to albumin-corrected calcium. Of these, 7 patients (6.3% of 110 patients) were identified with pseudohypercalcemia upon ionized calcium assessment.

**Table 1:** The Baseline characteristic of patients with MG.

	Monoclonal Gammopathy (n=110)
Sex	
Male [n (%)]	55 (50%)
Female [n (%)]	55 (50%)
Age (years)	60 ± 11.51
The Type of MIg	
IgA [n (%)]	30 (27.27%)
IgG [n (%)]	46 (41.82%)
IgD [n (%)]	6 (5.45%)
IgM [n (%)]	4 (3.64%)
Free Light Chains [n (%)]	23 (20.91%)
Biclonal Gammopathy [n (%)]	1 (0.91%)
The Specific Disease	
MM [n (%)]	68 (61.8%)
Other [n (%)]	42 (38.18%)

### Factors influencing serum total calcium

The patients with hypercalcemia were stratified into the actual hypercalcemia and pseudohypercalcemia groups based on their ionized calcium. In the actual hypercalcemia group, both ionized calcium and serum total calcium were elevated, whereas in the pesudohypercalcemia group, serum total calcium were elevated, with normal ionized calcium. This distinction was crucial for exploring the factors influencing serum total calcium between the two groups.

Compared with the actual hypercalcemia group and the pseudohypercalcemia group, the albumin level was higher in the actual hypercalcemia group than in the pseudohypercalcemia group (P <0.05), while the globulin level in the pseudohypercalcemia group was higher than in the actual hypercalcemia group (**Appendix Table E**). These two factors were included in the univariate analysis (**Appendix Table F**). We found that only globulin was a risk factor leading to pseudohypercalcemia in the population with hypercalcemia.

Recognizing hyperglobulinemia as a risk factor for pseudohypercalcemia, we utilized ROC curves to further analyze its diagnostic potential (**Appendix Figure C**). With a cutoff value of 61 g/L for globulin, the sensitivity for diagnosing pseudohypercalcemia was 83.3%, and the specificity was 76.9% (AUC=0.837, P < 0.05). This suggests that a globulin level of ≥61 g/L should raise suspicion for pseudohypercalcemia in hypercalcemia patients.

### Factors influencing bound calcium

In the nonhypercalcemia group, both ionized calcium and bound calcium were normal, whereas in the pesudohypercalcemia group, bound calcium were elevated, with normal ionized calcium. This study compared the pseudohypercalcemia group and the nonhypercalcemia group to identify the influencing factor of bound calcium.

Further analysis comparing the pseudohypercalcemia and the nonhypercalcemia groups revealed that albumin levels were significantly higher in the nonhypercalcemia group(*P <*0.05), while globulin levels were markedly higher in the pseudohypercalcemia group (**Appendix Table H**). Univariate analysis identified both albumin and globulin as potential risk factors influencing the bound calcium(**Appendix Table I**).Multiple logistic regression analysis confirmed both albumin and globulin as independent factors associated with pesudohypercalcemia(**Appendix Table J**, *P <*0.05).

### Formula establishment and validation

Given the influence of albumin and globulin on bound calcium, we developed a formula to correct ionized calcium levels, aiming to more accurately reflect physical calcium levels through multiple linear regression (**Table 2**). The formula is as follow:

**Table 2:** The multiple linear regression for albumin and globulin.

Item	B	Sig.	Collinearity Diagnostics	
			Tolerance	Variance Inflection Factor
Constant	0.163	0.022		
Serum Total Calcium	0.517	0.000	0.964	1.038
Albumin	-0.004	0.000	0.929	1.077
Globulin	-0.001	0.031	0.925	1.081


The Albumin and Globulin Corrected Ionized Calciummmol/L=0.517 × Serum Total Calcium (mmol/L)−0.04×Albumin (g/L)−0.001×Globulin (g/L)+0.163

The formula was validated using a paired sample t-test with data from 110 patients, demonstrating consistency with ionized calcium level measured by blood gas analysis (*P*=0.389). An additional 33 patients were enrolled to further verify the formula, with result aligning with blood gas test (*P*=0.160).

Comparing the new formula with the traditional albumin-corrected calcium, the albumin and globulin-corrected ionized calcium formula showed a sensitivity of93.75%, and a specificity of 96.85% for diagnosing hypercalcemia (**Table 3**), with the Youden index of 0.906. In contrast, the traditional formula had a sensitivity of 93.75% and a specificity of 94.49% (**Table 4**), yielding a Youden index of 0.88.

**Table 3:** The diagnosis of hypercalcemia by albumin and globulin-corrected ionized calcium.

		Ionized Calcium		N
		Hypercalcemia	Non-hypercalcemia	
Albumin and globulin-Corrected Ionized Calcium	Hypercalcemia	15	4	19
					Non-hypercalcemia	1	123	124
	N	16	127	143				

**Table 4:** The diagnosis of hypercalcemia by albumin-corrected ionized calcium.

		Ionized Calcium		N
		Hypercalcemia	Non-hypercalcemia	
Albumin-Corrected calcium	Hypercalcemia	15	7	22
	Non-hypercalcemia	1	120	121
	N	16	127	143

### Association of monoclonal immunoglobulin types with pseudohypercalcemia

**Figure 1:**
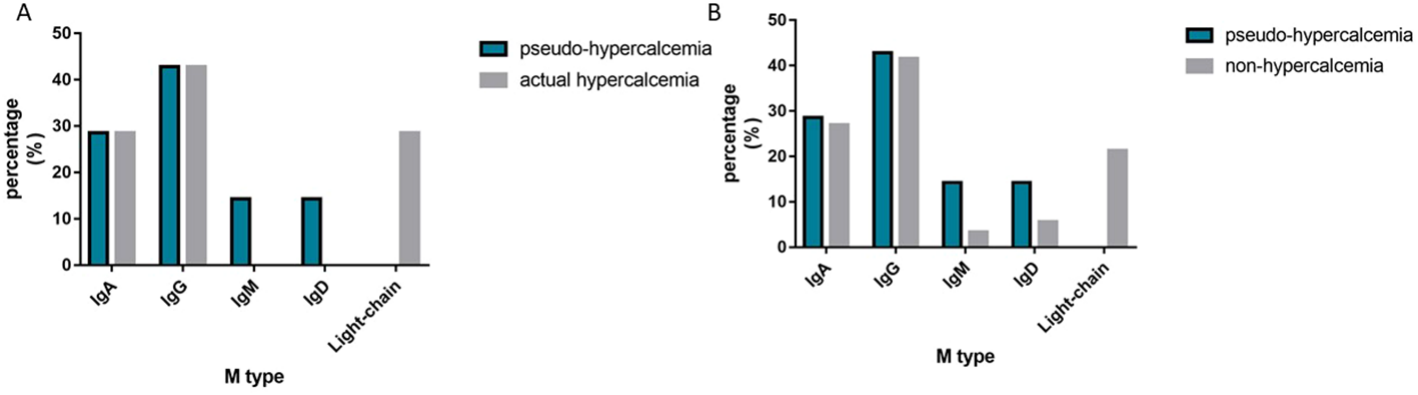
The Distribution of M-protein type. **(A)** shows the distribution of M-protein type between the pseudo-hypercalcemia group and the actual hypercalcemia group; **(B)** shows the distribution of M-protein type between the pseudo-hypercalcemia group and the non-hypercalcemia group.

Our study confirmed that globulin affects the bound calcium in patients with monoclonal gammopathy, corroborating previous studies that suggested calcium binding to monoclonal immunoglobulin (7, 23, 25, 35–40). Analyzing the association between the type of monoclonal immunoglobulin and pseudohypercalcemia revealed no significant differences in the distribution of IgA, IgG, IgM, and IgD types among the pseudohypercalcemia, actual hypercalcemia, and nonhypercalcemia groups. (P > 0.05, **Figure 1**). Interestingly, there were no cases for free light chains or biclonal immunoglobulin.

**Figure 2:**
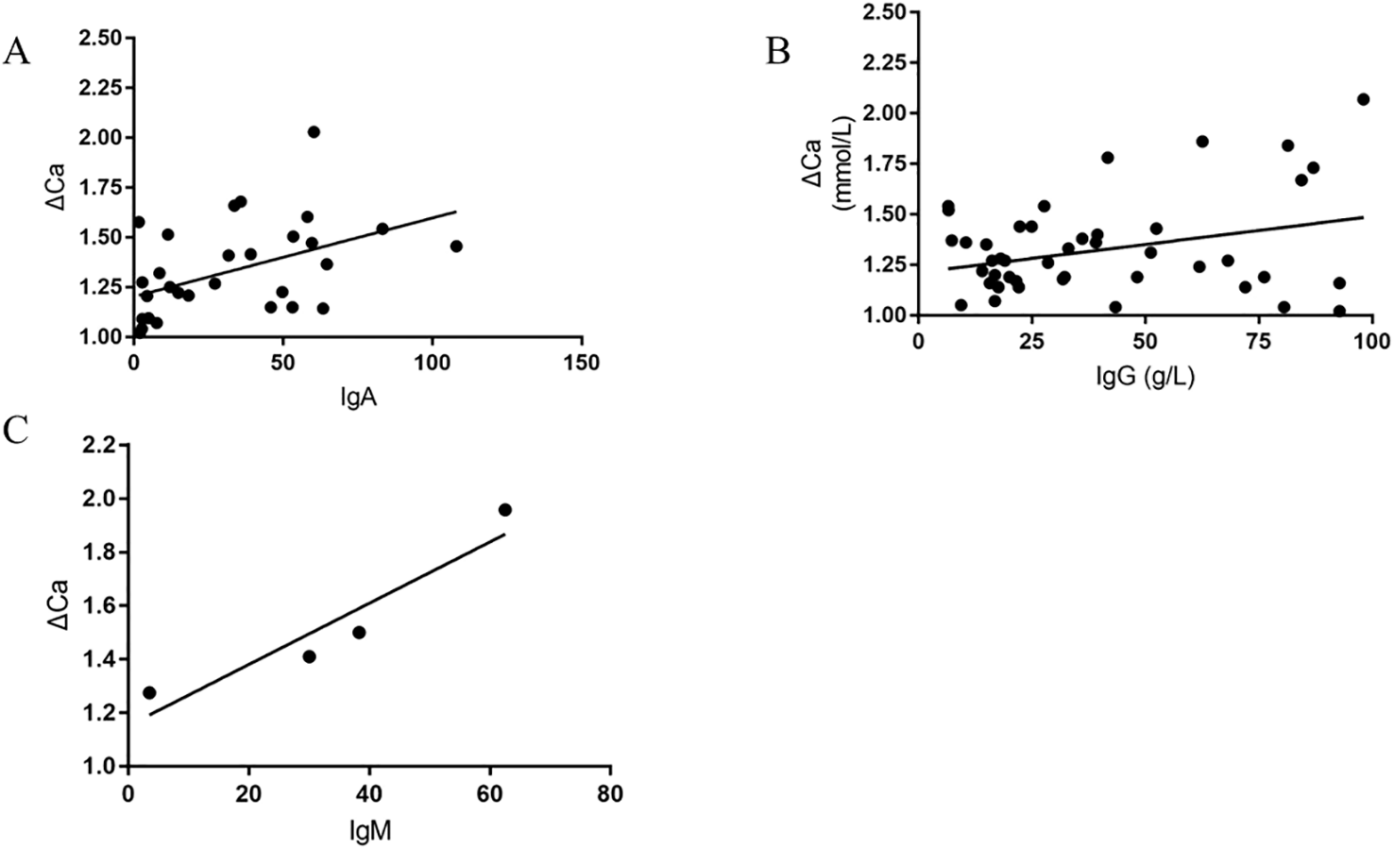
The Scatter plots of Bound calcium versus IgA, IgG, and IgM, respectively (Bound calcium≈ΔCa= serum total calcium minus ionized calcium, **(A)** shows The Scatter plots of Bound calcium versus IgA; **(B)** shows The Scatter plots of Bound calcium versus IgG; **(C)** shows The Scatter plots of Bound calcium versus IgM).

The high bound calcium, attributed to pseudohypercalcemia, are thought to bind with globulin. Some studies have indicated that monoclonal immuglobulin combined with calcium leads to pseudohypercalcemia. As a result, we verified the relationship between the bound calcium and monoclonal immuglobulin, finding a linear correlation between IgA/IgG and IgM with bound calcium (**Figure 2**).

## Discussion

Monoclonal gammopathy is a prevalent hematological disease, with hypercalcemia being a frequent clinical manifestation. Moderate-to-severe hypercalcemia is an urgent situation necessitating prompt intervention due to its life-threatening nature. Our study identified a subset of patients with markedly elevated corrected calcium who lacked the typical clinical signs of hypercalcemia, suggesting the presence of pseudohypercalcemia despite normal ionized calcium.

In our research, we found that 19% of patients with monoclonal gammopathy exhibited hypercalcemia, a proportion slightly higher than that observed in patients with multiple myeloma (12, 13). In addition, 6.3% of the patients were identified with pseudohypercalcemia. The aggressive treatment of hypercalcemia in these patients not only failed to address the clinical issues, but also imposed additional psychological and economic burden, complicating subsequent management. This underscores the critical need to discern the causes and characteristics of pseudohypercalcemia in patients with monoclonal gammopathy accurately.

The influence of globulin on calcium has been scarcely explored, with no existing studies on a globulin-corrected calcium formula. Our prospective study aimed to elucidate the relationship between calcium and globulin. We found that when globulin was ≥61g/L, the proportion of pseudohypercalcemia improved. Therefore, we hypothesized that this threshold could be a critical indicator for pseudohypercalcemia in hypercalcemia patients. This finding can be utilized to guide the use of blood gas tests or the application of the new formula for accurate diagnosis, which can minimize unnecessary blood gas analysis. Our result suggest that both albumin and globulin influence the bound calcium fraction, indicating that these proteins may modulate serum total calcium though calcium binding. The presence of globulin affects the albumin-corrected calcium. This indicates that this traditional correction may not be suitable for assessing the calcium level in patents with monoclonal gammopathy, where globulin is elevated. As a result, the use of the traditional albumin-corrected calcium formula in patients with monoclonal gammopathy) was restricted.

Therefore, we constructed a novel formula that corrects for both albumin and globulin to better align serum total calcium with ionized calcium. We verified this formula by comparing it with ionized calcium through a blood gas test. We found that the new formula has a good fit with ionized calcium. The Youden index of the new formula surpassed that of the traditional albumin-corrected calcium formula, indicating its potential superiority in diagnosing hypercalcemia. Moreover, the coefficients in the new formula of albumin and globulin are close to the ratio of albumin and globulin binding to bound calcium, which are 81% and 19%, respectively (21).

Our analysis revealed that patients with pseudohypercalcemia exhibited a variety of non-free light chain monoclonal immunoglobulins. There were no significant differences among the types of non-free light chain monoclonal immunoglobulins. This finding implies that the free light chain of monoclonal Iimmuglobulins do not lead to pseudohypercalcemia, as calcium binding is restricted to the Fab segment of the monoclonal immuglobulin molecule (38). Since the Fab segment is composed of light chains and some heavy chains, neither light chains monoclonal immunoglobulin nor heavy chains monoclonal immunoglobulin alone can bind to calcium, resulting in pseudohypercalcemia.

This study addresses a common clinical dilemma: the potential misdiagnosis of hypercalcemia in patients with pseudohypercalcemia, leading to unnecessary aggressive treatment. This management cannot address the clinical issues, and adverse reactions caused by treatment to reduce calcium may occur. Many studies have shown that hypercalcemia is associated with a poor prognosis in many diseases (29, 44–47). Misdiagnosing patients with pseudohypercalcemia as hypercalcemia may have a significant impact on disease diagnosis, prognostication, therapeutic choice, and the physical and psychological burden on patients. The standard method for differentiating actual hypercalcemia from false hypercalcemia is ionized calcium detected by blood gas analysis. However, this detection method is limited in clinical application, and improper sample processing and test delivery time have a serious impact on the detection results of ionized calcium. Our novel formula, based on accessible clinical indices, aims to assist physicians in assessing the actual ionized calcium of patients, thereby reducing unnecessary interventions and improving diagnostic accuracy and therapeutic decision-making. Finally, the psychological, physiological and economic burdens on patients can be relieved.

While our study presents a significant advancement in the diagnosis of hypercalcemia in patients with monoclonal gammopathy, it is not without limitations. The need for a larger sample size to refine the formula and further validate its scientific and objective applicability is recognized. The absence of corroborating studies necessitates additional research to confirm our findings. Future work should also explore the applicability of our formula in normal populations and patients with non-monoclonal gammopathy conditions.

## Conclusion

This study provides compelling evidence that elevated globulin significantly affects serum total calcium, potentially resulting in pseudohypercalcemia. Our findings underscore the importance of considering pseudohypercalcemia in the differential diagnosis of hypercalcemia, particularly when globulin levels are ≥ 61 g/L in patients. This threshold offers a practical clinical guideline for further investigation and assessment of calcium status. Given the influence of albumin and globulin on bound calcium, we developed a formula to correct ionized calcium levels. And the new formula surpassed the traditional albumin-corrected calcium formula through validation. Our research also highlights that the occurrence of pseudohypercalcemia is not exclusive to any specific type of non-free light chain monoclonal immunoglobulin, suggesting a common underlying mechanism. Neither light chains monoclonal immunoglobulin nor heavy chains monoclonal immunoglobulin alone can result in pseudohypercalcemia.

## Data Availability

The raw data supporting the conclusions of this article will be made available by the authors, without undue reservation.
