# Supplementary material for: The identification and correction of pseudohypercalcemia
Source: Front Oncol. 2024 Oct 31;14:1441851. doi: 10.3389/fonc.2024.1441851 (PMC11560909; doi:10.3389/fonc.2024.1441851)
Supplement: Supplementary file 1 [file Table1.docx]

**Appendix Table A. The case reports of pseudo-hypercalcemia**

| **The** | **Author** | **Diagnosis** | **Case** | **Serum total** | **Ionized calcium** |
| --- | --- | --- | --- | --- | --- |
| **type** |  |  |  |  |  |
| **of** |  |  |  | **calcium** |  |
| **MIg** |  |  |  |  |  |
| IgG | Folke | MM | 1 | 7.2meq/Lite | 2.02meq/Lite |
|  | Lindgarde (38) |  |  | (4.5~5.3meq/Lite) | (1.95~2.41meq/Lite) |
| IgM | L Side (32) | WM | 1 | 3.01 mmol/L | 1.27mmol/L |
| IgA | C J | MM | 1 | 3.5mmol/L | 1.29mmol/L |
|  | Pearce (37) |  |  |  |  |
| IgM | Rhys | WM | 1 | 3.02mmol/L | 1.15mmol/L |
|  | John (36) |  |  |  |  |
| IgG | GiamPaolo | MM | 1 | 14.6mg/dL | 4.8mg/dL |
|  | Merlini (35) |  |  |  |  |
| IgG | Jeffrey P. | MM | 1 | 17.8mg/dL | 5.2mg/dL |
|  | Jaffe (34) |  |  |  |  |
| IgM | Denise | Cryoglobulinema | 1 | 2.98mmol/L | 1.27mmol/L |
|  | Tong (33) |  |  |  |  |

**The diagnose standard of hypercalcemia** ：Serum total calcium≥5.3meq/Lite or≥2.75mmol/L or≥11mg/dL；Ionized calcium≥2.41meq/Lite or≥1.33mmol/L or≥5.33mg/dL.

**Appendix Table B. The multivariate Logistic analysis for the groups of hypercalcemia and non-hypercalcemia**

|  | **OR** | **95%*CI*** | ***P*-Value** |
| --- | --- | --- | --- |
| Crea（μmol/L） | 1.002 | 1.001 ~ 1.004 | 0.009* |
| Albumin（g/L） | 0.956 | 0.900 ~ 1.016 | 0.148 |
| Globulin（g/L） | 1.026 | 1.010 ~ 1.043 | 0.001* |
| Bone destruction | 1.654 | 0.525 ~ 5.209 | 0.390 |
| Disease for MM | 0.967 | 0.279 ~ 3.354 | 0.958 |

**Appendix Table C. The distribution of calcium in three groups**

|  | **Serum total Calcium** | **Bound Calcium** | **Ionized Calcium** |
| --- | --- | --- | --- |
| **The pseudohypercalcemia group** | High | High | Normal |
| **The Actual hypercalcemia group** | High | Normal or High | High |
| **The Non-hypercalcemia**  **Group** | Normal | Normal | Normal |

**Appendix Table D. The result of the diagnosis of hypercalcemia by calcium corrected by**

**formula**

|  |  | **Ionized Calcium** | |
| --- | --- | --- | --- |
|  |  | **Hypercalcemia** | **Non-Hypercalcemia** |
| Calcium  Corrected by | Hypercalcemia | True Positive | False Positive |
|  | Non-Hypercalcemia | False negative | True negative |

**Appendix Table E. Analysis of the Actual Hypercalcemia Group and the**

**Pseudo-hypercalcemia group**

|  | **Actual Hypercalcemia**  **Group (n=14)** | **Pseudo-hypercalcemia Group (n=7)** | ***P*-value** |
| --- | --- | --- | --- |
| Sex |  |  |  |
| Male [Cases (%)] | 5 (35.7%) | 4 (57.1%) | 0.397 |
| Female [Cases (%)] | 9 (64.3%) | 3 (42.9%) |  |
| Age (years) | 69.93±10.27 | 57.57±14.59 | 0.094 |
| The Specific Disease |  |  |  |
| MM [ Cases (%)] | 11 (78.6%) | 6 (85.7%) | 0.694 |
| Others [Cases (%)] | 3 (21.4%) | 1 (14.3%) |  |
| The Type of Mig (Free Light Chains) |  |  |  |
| No [Cases (%)] | 10 (71.4%) | 7 (100%) | 0.255 |
| Yes [Cases (%)] | 4 (28.6%) | 0 (0%) | 0.128 |
| Crea (μmol/L) | 192.5 (95.5 ~ 350.5) | 110.29±41.08 |  |
| Albumin (g/L) | 33.01±6.401 | 25.9 (21.5 ~ 26.7) | 0.025* |
| Globulin (g/L) | 52.55 (25.53 ~ 61.35) | 76.04±18 | 0.012* |
| Bone Destruction |  |  |  |
| Yes [Cases (%)] | 10 (71.4%) | 4 (57.1%) | 0.516 |
| No [Cases (%)] | 4 (26.6%) | 3 (42.9%) |  |

**Appendix Table F. Univariate Logistic Analysis between the Group of Actual hypercalcemia**

**and Pseudo-hypercalcemia**

|  | **Actual hypercalcemia** | **Pseudo-hypercalcemia** | **OR** | ***P-*Value** |
| --- | --- | --- | --- | --- |
|  | **Group（n=14）** | **Group（n=7）** |  |  |
| Albumin (g/L) | 33.01±6.401 | 25.9 (21.5 ~ 26.7) | 1.186 | 0.083 |
| Globulin (g/L) | 52.55 (25.53 ~ 61.35) | 76.04±18 | 0.928 | 0.039* |

**Appendix Table H. The comparison between the pseudo-hypercalcemia group and the non-hypercalcemia group**

|  | The  Non-hypercalcemia  Group （n=89） | | The  Pseudo-hypercalcemia  Group（n=7） | P-Value |
| --- | --- | --- | --- | --- |
| Sex | |  |  |  |
| Male [Cases (%)] | | 46 (51.7%) | 4 (57.1%) | 0.78 |
| Female [Cases (%)] | | 43 (48.3%) | 3 (42.9%) |  |
| Age (years) | | 58.62±10.76 | 57.57±14.59 | 0.81 |
| The Specific Disease | |  |  |  |
| MM [Cases (%)] | | 51 (57.3%) | 6 (85.7%) | 0.235 |
| Other [Cases (%)] | | 38 (42.7%) | 1 (14.3%) |  |
| The Type of Mig  (Free Light Chains) | |  |  |  |
| No [Cases (%)] | | 70 (78.7%) | 7 (100%) | 0.339 |
| Yes [Cases (%)] | | 19 (21.3%) | 0 (0%) |  |
| Crea (μmol/L) | | 86 (64.5 ~ 151) | 110.29±41.08 | 0.477 |
| Albumin (g/L) | | 37 (31.1 ~ 40) | 25.9 (21.5 ~ 26.7) | 0.006* |
| Globulin (g/L) | | 35 (25 ~ 54) | 76.04±18 | 0.001* |
| Bone Destruction | |  |  |  |
| Yes [Cases (%)] | | 37 (41.6%) | 4 (57.1%) | 0.456 |
| No [Cases (%)] | | 52 (58.4%) | 3 (42.9%) |  |

**Appendix Table I. Univariate Logistic Analysis between the Group of Non-hypercalcemia and Pseudo-hypercalcemia**

|  | **The** | **The** | **OR** | ***P*-Value** |
| --- | --- | --- | --- | --- |
|  | **Non-hypercalcemia** | **Pseudo-hypercalcemia** |  |  |
|  | **Group（n=89）** | **Group (n=7)** |  |  |
| Albumin (g/L) | 37 (31.1 ~ 40) | 25.9 (21.5 ~ 26.7) | 0.862 | 0.003* |
| Globulin (g/L) | 35 (25 ~ 54) | 76.04±18 | 1.055 | 0.002* |

**Appendix Table J. The Mutiple Logistic Regression Analysis between the Group of Non-hypercalcemia and Pseudo-hypercalcemia**

|  | **OR** | **95%*CI*** | ***P* -Value** |
| --- | --- | --- | --- |
| Albumin (g/L) | 0.815 | 0.693 ~ 0.959 | 0.014* |
| Globulin (g/L) | 1.063 | 1.020 ~ 1.109 | 0.004* |
