# Supplementary figures and images for: The identification and correction of pseudohypercalcemia
Source: Front Oncol. 2024 Oct 31;14:1441851. doi: 10.3389/fonc.2024.1441851 (PMC11560909; doi:10.3389/fonc.2024.1441851)

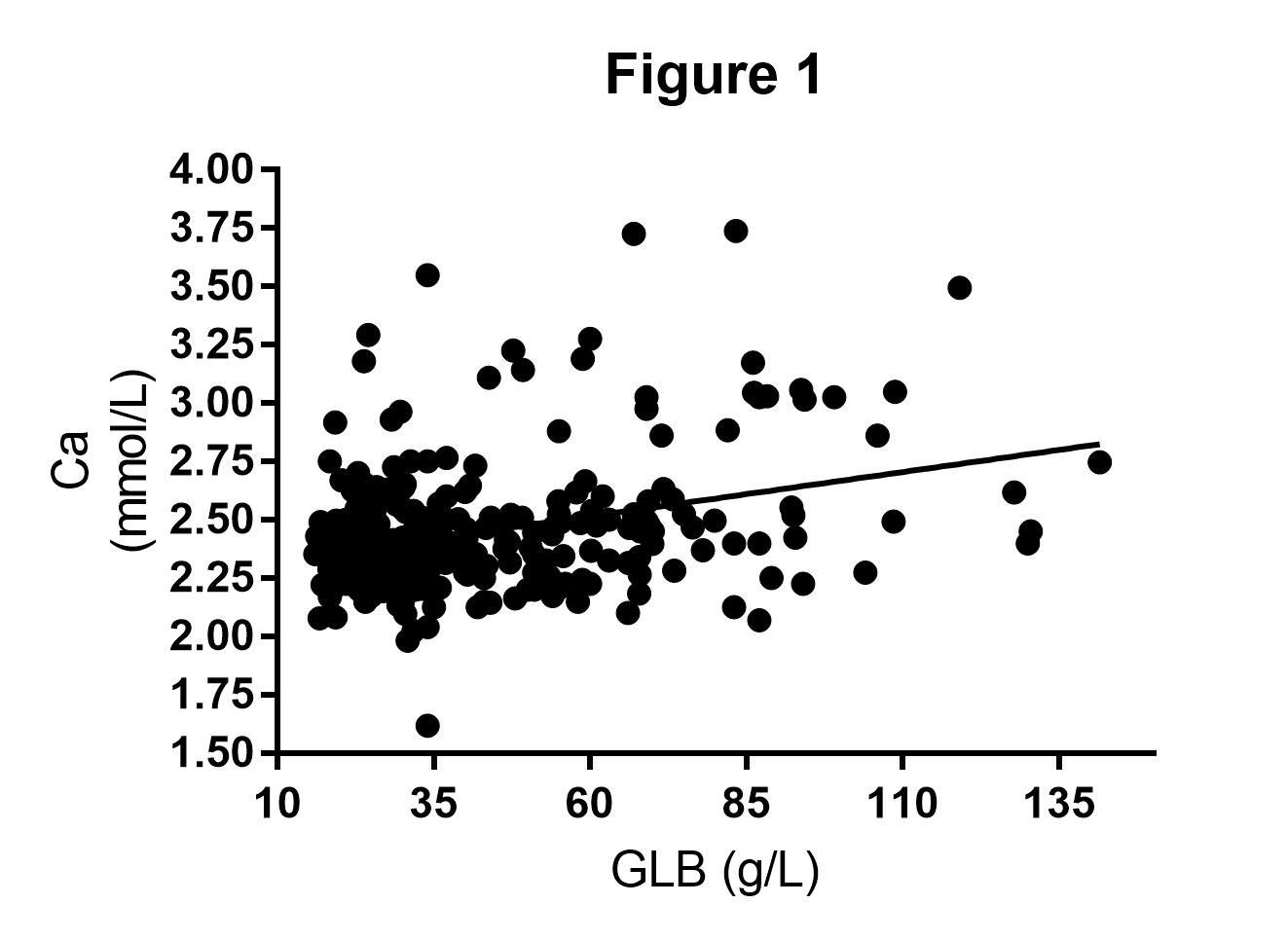

Supplement: Supplementary file 2 [file Image1.jpeg]

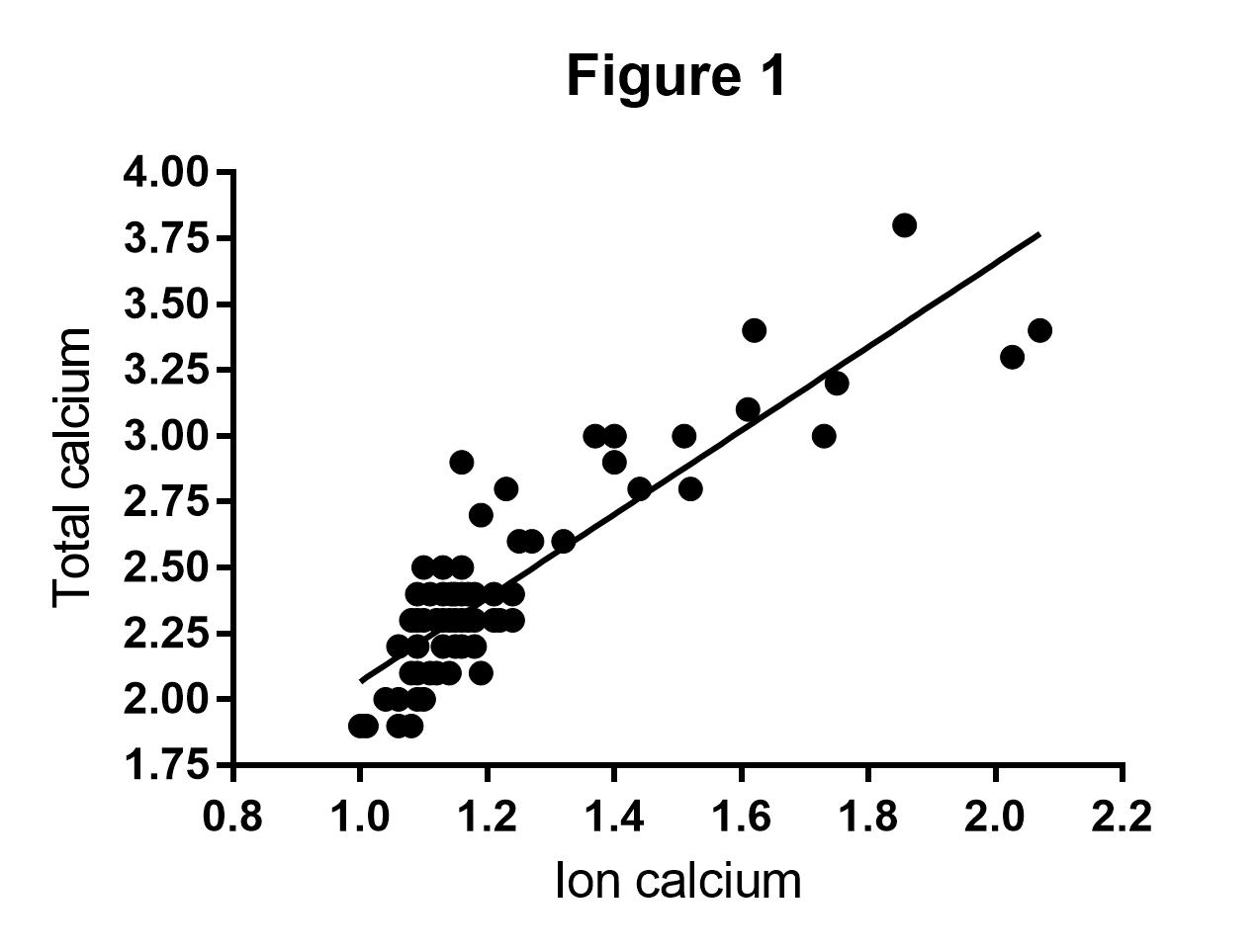

Supplement: Supplementary file 3 [file Image2.jpeg]

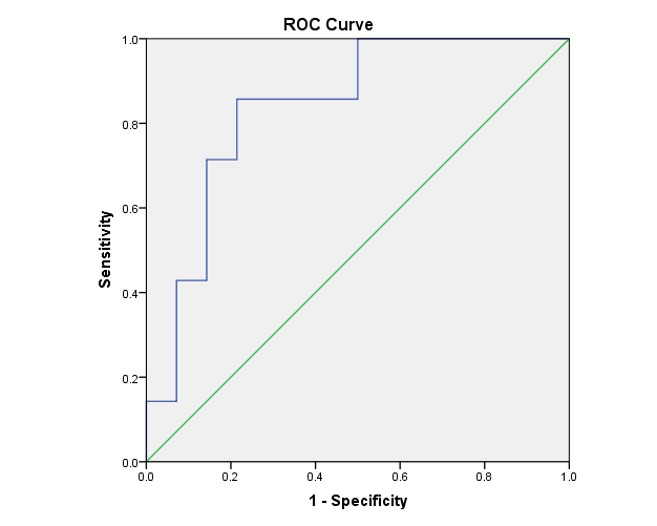

Supplement: Supplementary file 4 [file Image3.jpeg]
